# The *Moraxella catarrhalis* AdhC–FghA system is important for formaldehyde detoxification and protection against pulmonary clearance

**DOI:** 10.1007/s00430-024-00785-0

**Published:** 2024-03-06

**Authors:** Dina Othman, Noha M. Elhosseiny, Wafaa N. Eltayeb, Ahmed S. Attia

**Affiliations:** 1 Graduate Program, Department of Microbiology and Immunology, Faculty of Pharmacy, Cairo University, Cairo, 11562 Egypt; 2 Department of Microbiology and Immunology, Faculty of Pharmacy, Cairo University, Room #D404, Kasr El-Ainy Street, Cairo, 11562 Egypt; 3 Department of Microbiology, Faculty of Pharmacy, Misr International University, Cairo, 19648 Egypt

**Keywords:** *Moraxella catarrhalis*, Formaldehyde resistance, *S*-hydroxymethyl alcohol dehydrogenase, *S*-formyl glutathione hydrolase, Pulmonary clearance

## Abstract

Multidrug-resistant clinical isolates of *Moraxella catarrhalis* have emerged, increasing the demand for the identification of new treatment and prevention strategies. A thorough understanding of how *M. catarrhalis* can establish an infection and respond to different stressors encountered in the host is crucial for new drug-target identification. Formaldehyde is a highly cytotoxic compound that can be produced endogenously as a by-product of metabolism and exogenously from environmental sources. Pathways responsible for formaldehyde detoxification are thus essential and are found in all domains of life. The current work investigated the role of the system consisting of the *S*-hydroxymethyl alcohol dehydrogenase (AdhC), a Zn-dependent class III alcohol dehydrogenase*,* and the *S*-formyl glutathione hydrolase (FghA) in the formaldehyde detoxification process in *M. catarrhalis*. Bioinformatics showed that the components of the system are conserved across the species and are highly similar to those of *Streptococcus pneumoniae*, which share the same biological niche. Isogenic mutants were constructed to study the function of the system in *M. catarrhalis*. A single *fghA* knockout mutant did not confer sensitivity to formaldehyde, while the *adhC–fghA* double mutant is formaldehyde-sensitive. In addition, both mutants were significantly cleared in a murine pulmonary model of infection as compared to the wild type, demonstrating the system’s importance for this pathogen’s virulence. The respective phenotypes were reversed upon the genetic complementation of the mutants. To date, this is the first study investigating the role of the AdhC–FghA system in formaldehyde detoxification and pathogenesis of *M. catarrhalis*.

**Supplementary Information:**

The online version contains supplementary material available at 10.1007/s00430-024-00785-0.

## Introduction

*Moraxella catarrhalis*, previously considered a commensal microorganism, has been commonly implicated as the major etiological agent of otitis media and sinusitis in children, and in exacerbation of chronic obstructive pulmonary disease in adults [1]. Multidrug-resistant clinical isolates of *M. catarrhalis* have emerged, increasing the demand for the identification of new treatment and prevention strategies against this pathogen, especially in the absence of an efficient vaccine [2, 3].

A formaldehyde detoxification system is essential for microorganisms to protect themselves from cytotoxic formaldehyde [4]. In the human body, formaldehyde is produced during the metabolism of methanol, adrenaline, creatine, and histones. Most importantly, it is a by-product of some chemical reactions associated with the immune response, such as the methylation of histamine. During the respiratory burst by macrophages and neutrophils to kill pathogens, superoxide and hydrogen peroxide are generated, which in turn react with bacterial iron-sulfur clusters to produce free radicals. These free radicals react with sugars to produce formaldehyde as a toxic end product that could be used to fight invading pathogens [4]. Formaldehyde is also produced as a part of bacterial metabolism in cell biological processes [5]. For all of the aforementioned reasons, the formaldehyde detoxification processes are required to avoid formaldehyde lethal and mutagenic effect [6].

Formaldehyde detoxification is achieved through three mechanisms; thiol-dependent, ribulose monophosphate-dependent, and pterin-dependent mechanisms [4]. The glutathione-dependent repair system, also known as the thiol-dependent pathway, appears to be widely spread in nature and has been found in most prokaryotes, and all eukaryotes [7]. In the majority of microorganisms, the thiol is the tripeptide glutathione. Initially, glutathione binds to formaldehyde to form *S*-hydroxymethylglutathione [8]. This reaction occurs spontaneously in most microorganisms, with some exceptions where this reaction is catalyzed by a glutathione-dependent formaldehyde-activating enzyme, Gfa [9, 10]. The *S*-hydroxymethylglutathione adduct is then oxidized by a zinc-containing, nicotinamide adenine dinucleotide (NAD^+^)-dependent alcohol dehydrogenase, AdhC, to generate the thioester *S-*formylglutathione [11]. Finally, formate is produced and glutathione is regenerated upon the hydrolysis of S-formyl glutathione. This last step is catalyzed by an esterase, EstD, or *S*-formylglutathione hydrolase, FghA [12].

Numerous studies have been investigating the genetic factors involved in formaldehyde detoxification and their role in stress protection, bacterial virulence, and biofilm formation in different microorganisms [6, 7, 13–19]. For example, the glutathione-dependent formaldehyde detoxification system AdhC–EstD was shown to be important for the optimum viability of *Neisseria meningitidis* in biofilm communities [20]. In another study, the loss of the encoding gene of EstD in *N. gonorrhoeae* caused an impairment in the ability of this organism to survive within human cervical epithelial cells [17]. While many of the genetic factors contributing to the physiology and virulence of *M. catarrhalis* have been identified [21–23], the role of formaldehyde detoxification in these processes has not been yet investigated. In this study, the *adhC* and *fghA* genes of *M. catarrhalis* have been investigated with respect to their potential role in the formaldehyde detoxification and the virulence of this pathogen.

## Materials and methods

### Ethics statement

Animal procedures were approved by the Research Ethics Committee of the Faculty of Pharmacy, Cairo University, Approval No. MI (2510), following the Guide for the Care and Use of Laboratory Animals published by the Institute of Laboratory Animal Research, USA.

### Bacterial strains and culture conditions

*M. catarrhalis* O35E was kindly provided by Dr. Eric J. Hansen [24] and it was used as the wild type (WT). The derivatives were all generated in its background. *M. catarrhalis* strains were grown on Tryptic Soy Agar (TSA) or Columbia blood agar at 37 °C and 5% CO_2_ in a carbon dioxide incubator (Binder, Germany), or in Tryptic Soy Broth (TSB) at 37 °C with shaking at 180 rpm under aerobic conditions [25]. *Escherichia coli* DH5-α, used as a cloning host, was grown at 37 °C in TSB with shaking at 180 rpm, or on TSA. When needed, media were supplemented with kanamycin at a final concentration of 15 µg/mL, streptomycin at a final concentration of 250 µg/mL, and ampicillin at a final concentration of 100 μg/mL.

### Bioinformatics analyses

The protein sequence of the EstD of *N. meningitidis (*NCBI protein id CAM08666.1) served as a template for a BlastP analysis [26], to identify homologs of this esterase in *M. catarrhalis* O35E. To survey the conservation of the protein, the BlastP search was also extended to all the strains available in *M. catarrhalis* taxid 480. Sequences of previously studied FghA homologs were retrieved from the NCBI (Table S1), and the same was performed with the AdhC homologs (Table S2). Then, multiple sequences’ alignment with the Blast-retrieved FghA of *M. catarrhalis* O35E (NCBI Protein id EGE27440.1) as a query sequence was carried on using Clustal Omega [27] applyling the default parameters. Phylogenetic trees were constructed via NGphylogeny.fr web tool, which employs multiple alignment using fast Fourier transform (MAFFT) for multiple sequence alignment, Block Mapping and Gathering with Entropy (BMGE) for alignment curation, Phylogeny software for the maximum-likelihood principle (PhyML) for tree inference, and finally Newick display for tree rendering [28–32]. To calculate the percent similarity and identity, EMBOSS Needle tool was used with the default parameters [33]. To further confirm the interaction between AdhC and FghA and show possible interactions with other proteins, the protein–protein functional interaction analysis tool STRING [34] was used. To investigate if the *adhC* and *fghA* genes form an operonic pair as consistently reported for the system, the operon prediction tool Operon Mapper [35] was used applying the default parameters, and the whole *M. catarrhalis* O35E genome (AERL00000000.1) as a query sequence.

### Construction of *M. catarrhalis ΔfghA* and *ΔadhC–fghA* deletion mutants and rescue strains

**Fig. 1:**
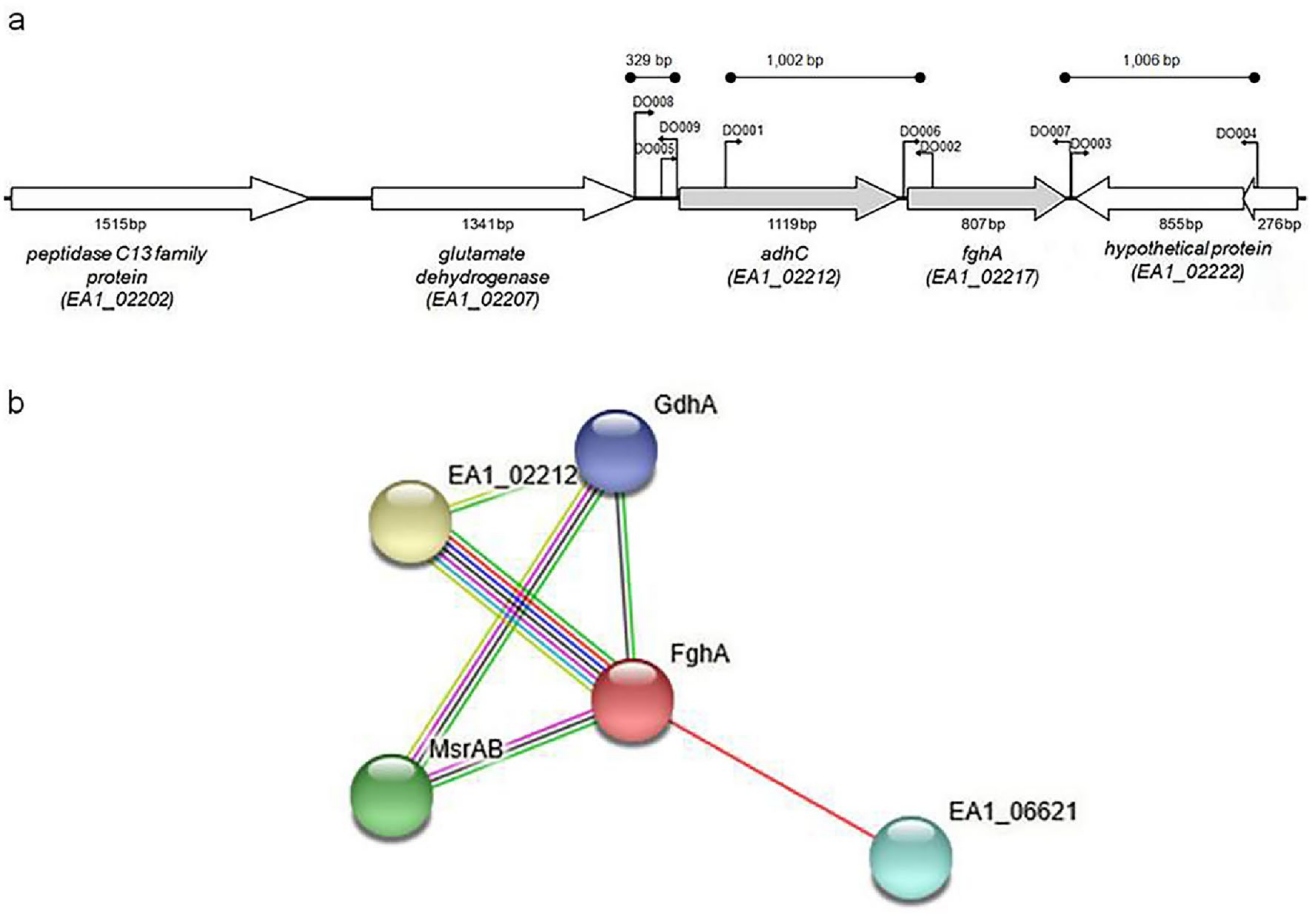
Genetic organization and functional relation between the *M. catarrhalis adhC* and *fghA*. **A** A schematic diagram showing the organization of the neighboring ORFs in the genetic loci of the *adhC* and *fghA* in the *M. catarrhalis* WT O35E genome. The binding position of the primers used in this study and the loci tags are indicated. The direction of the ORF arrows indicates the direction of transcription. The ruler above the arrows indicates the size of DNA fragment in base pairs; bp. The map was generated by Ankh diagram v1.1tool by HITS Solutions Co. (Bioinformatics Department, Cairo, Egypt). **B** A schematic diagram representing the interaction between AdhC (encoded by EA1_02212) with FghA and other *M. catarrhalis* proteins including Gdsl-like lipase (encoded by EA1_06621), MsrAB, and GdhA. The figure was generated using STRING database and the lines drawn between the functional pairs represent the predicted functional relationship (neighborhood; green, gene fusion; red, co-occurrence; dark purple, co-expression; black, databases; teal, text mining; yellow, experimentally determined interactions; pink line, and protein homology; light blue)

Using the *M. catarrhalis* O35E chromosomal DNA as a template, the primer pairs DO001–DO002 and DO003–DO004 (Table S3 and Fig. 1) were used to amplify a 1002 and a 1006 base pair fragments upstream and downstream of the *fghA* open-reading frame (ORF), respectively. The fragments were then digested using XmaI and ligated together. The ligation product was used as a template for a second PCR reaction using primer pair DO001–DO004. The product was then ligated into the rapid cloning vector pJET1.2/blunt (Thermo Fisher Scientific, Lithuania), to yield plasmid pJET-U + D. A non-polar kanamycin resistance cassette was obtained by digesting plasmid pUC18K [36] with XmaI, followed by gel purification. The product was then ligated with plasmid pJET-U + D digested with the same restriction enzyme, then transformed into *E. coli* DH5-α and plated on TSA containing ampicillin and kanamycin. The resultant plasmid was designated pJET-UkanD. This plasmid was used to amplify a ~ 3 kb construct consisting of the kanamycin cassette flanked by the *fghA* upstream and downstream fragments, and the product was transformed into *M. catarrhalis* O35E as previously described [37]. The homologous recombination of the mutant construct into the chromosome and the replacement of the WT *fghA* gene to yield *ΔfghA* mutant was confirmed by a series of PCR reactions using primers within and outside the mutant construct. To construct the *ΔadhC-fghA* double mutant, a similar approach was adopted but using primer pairs DO008–DO009 and DO003–DO004 to amplify a 329 and a 1002 base pair fragments upstream and downstream of *adhC* and *fghA*, respectively. To repair the *ΔfghA* mutant, the fragment amplified with primer pair DO001–DO004 from the WT O35E *M. catarrhalis* strain together with the mutated *rpsL* amplicon [38] were transformed into the *ΔfghA* in a congression experiment as previously described [39]. Isolated colonies that could grow on streptomycin, and failed to grow on kanamycin, were selected as potential complemented mutants, confirmed using PCR, and designated *ΔfghA/R*. To repair *ΔadhC–fghA* mutant, a similar approach was used, but using primer pair DO008–DO004 to amplify the WT amplicon. The confirmed rescue mutant was designated *ΔadhC–fghA/R*. The sequences of all the oligonucleotides used in this study are listed in Table S3.

### Growth curve analysis

Colonies of the WT O35E, *ΔfghA, ΔfghA*/*R, ΔadhC-fghA,* and *ΔadhC-fghA*/*R* grown overnight on Columbia blood agar were suspended in TSB to an optical density at 600 nm (OD_600_) ~ 1.0 then diluted 1:50 in 15 mL TSB broth. The cultures were incubated in a shaking incubator at 37 °C and 180 rpm and the OD_600_ was measured each hour for 8 h using a visible spectrophotometer Jenway 6300 (Jenway, United Kingdom). Growth curves were constructed by plotting OD_600_ versus time.

### Formaldehyde sensitivity assay

Formaldehyde susceptibility was assessed using the disc diffusion susceptibility assay as previously described [20]. Briefly, cells of the WT O35E, *ΔfghA, ΔfghA*/*R, ΔadhC-fghA,* and *ΔadhC-fghA*/*R* freshly streaked on Columbia blood agar were suspended in TSB to an OD_600_ ~ 0.4. The adjusted cell suspensions were spread over TSA plates (supplemented with kanamycin for *ΔfghA* and *ΔadhC-fghA* mutants, streptomycin for *ΔfghA*/*R* and *ΔadhC-fghA*/*R* complemented mutants) using sterile cotton swabs. Sterile single Whatmann no. 1 filter paper discs were saturated with 5 μL of a 5% formaldehyde solution (Piochem, Egypt) and placed onto the agar surface. The diameter of the inhibition zone around the disc was measured after an overnight incubation at 37 °C in a CO_2_ incubator with the petri-dishes inverted.

Formaldehyde sensitivity was also tested by another assay [20]. Briefly, a bacterial suspension was prepared as in the sensitivity assay detailed above, and seven tenfold serial dilutions were prepared in a 96-well microplate in TSB. Five microliters of each dilution were spotted on TSA plates, supplemented with 0-, 0.8-, or 1-mM formaldehyde. The spots were left to dry then survival was determined by counting the number of visible colonies after an overnight incubation at 37 °C in a CO_2_ incubator with the petri-dishes inverted.

### Determination of the minimum inhibitory concentration (MIC)

To obtain a more quantitative assessment of the susceptibility of the five strains under investigation to formaldehyde, we performed an MIC experiment using the standard microdilution method [40]. Briefly, a 0.5 McFarland standard suspension of each of the five strains was prepared using freshly grown bacterial cells, and then, it was diluted 1:10 and 10 µL were used to inoculate 12 wells containing 190 µL of TSB containing twofold dilutions of formaldehyde from 1 mM.to 0.5 µM.

The wells incubated for 24 h at 37 °C then inspected for visual growth. The formaldehyde concentration in the first clear well was considered the corresponding MIC value for the respective strain.

### Protein profiling using sodium dodecyl sulfate-polyacrylamide gel electrophoresis (SDS-PAGE)

Cells of the five tested strains were grown overnight in TSB in the presence and absence of 1 mM formaldehyde in a shaking incubator, harvested by centrifugation, and resuspended in sterile saline to an OD_600_ ~ 1. The cell suspensions were mixed with a 3 × reducing Laemmli buffer [41]. These samples were then heated at 95 °C for 10 min in a thermal cycler (Boeco, Germany) before loading on a 10% SDS-PAGE gel. Gels were afterwards stained with Coomassie blue [41], visualized, and photographed using a gel documentation system (UVP, Germany).

### Murine pulmonary clearance model

The pulmonary clearance model in mice was carried out as previously described [42]. Briefly, five groups (*n* = 6) of 6–8-week-old female BALB/C mice were infected intranasally by injecting 40 μl of a bacterial suspension (∼5 × 10^6^ CFU) of each of the WT O35E, *ΔfghA, ΔfghA*/*R, ΔadhC-fghA,* and *ΔadhC-fghA/R* into the nostrils under anesthesia using 250 µL of 25 µg/mL 2,2,2-tribromoethanol. Four-and-a-half-hour post-inoculation, mice were sacrificed by an overdose of the anesthesia (750 µL of 25 µg/mL 2,2,2-tribromoethanol), followed by cervical dislocation. The lungs were excised, homogenized, serially diluted, and plated on TSB agar. Plates were incubated for 48 h followed by colony counting.

## Results

### *M. catarrhalis* possesses an EstD homolog

Using the *N. meningitidis* EstD as a query, Blastp against the *M. catarrhalis* O35E proteome returned a 268 amino acid protein (EGE27440.1) annotated as FghA (for *S*-formylglutathione hydrolase A) as the closest match, with a query coverage of 96%, and an identity of 54% (Fig. S1). Results of the NCBI BlastP between FghA of *M. catarrhalis* strain O35E (EGE27440.1) and other *M. catarrhalis* strains (taxid 480) shows a high degree of conservation with a percent identity range of 91.79–100% (Fig. S2). Alignment of homologs from different microbial genera, and even higher eukaryotes showed that all had high identity (40–65%) and similarity (55–77.7%) with the FghA of *M. catarrhalis* (Fig. S3 and Table S1). The highest similarity obtained was with *S. pneumoniae* (77.7%). Interestingly, although phylogenetic analysis showed that the *S. pneumoniae* FghA is still the closest evolutionary relative to the *M. catarrhalis* protein, those from closely related genera which scored the highest similarity such as *Neisseria*, and *Haemophilus* clustered together in a more distant branch of the tree. Meanwhile, the FghA homolog from *Paraccoccus* showed a closer relationship to the *M. catarrhalis* FghA, although it scored less on sequence similarity than the aforementioned species (Fig. S4).

We noticed that the ORF upstream of the *M. catarrhalis fghA* (Fig. 1A) was annotated as *adhC*, a gene encoding for *S*-hydroxymethyl alcohol dehydrogenase, and which usually forms an operon with *fghA* as previously reported [14, 20, 43]. To investigate the relationship between the two ORFs, first, the operon prediction tool Operon Mapper was used, and the results obtained demonstrated that *adhC* and *fghA* are expected to form an operon by a high probability of 0.97. Next, the protein interaction network database, STRING, was used to investigate if a predicted functional link between the two proteins is likely. The generated interaction network indicated that the *M. catarrhalis* AdhC and FghA are strongly predicted as functional partners by a score of 0.999. The two genes, Adhc and FghA, interact by gene neighborhood, gene fusion, gene co-occurrence, co-expression, and protein homology (Fig. 1B). The network also revealed the interaction by gene fusion between FghA with EA1_06621, a Gdsl-like lipase/ccyl hydrolase family protein. Another interaction by gene neighborhood between FghA and MsrAB (EA1_03390), a trifunctional thioredoxin/methionine sulfoxide reductase a/b protein was shown by the network. The final interaction by gene neighborhood and co-expression was predicted with GdhA (EA1_02207) an NADP-specific glutamate dehydrogenase and FghA. It is worth mentioning that while the confidence score of these former interactions was relatively low (0.4), the GdhA was the only protein predicted to interact with both FghA, and AdhC. Although it lies upstream of *adhC*, it was not predicted to form an operon with the pair.

A similar blast analysis to that conducted using the FghA was done using the *M. catarrhalis* O35E AdhC protein sequence. There was a high identity (41–81.7%) and similarity (49.2%-90.9%) between AdhC of *M. catarrhalis* and its homologs in other species (Fig. S5 and Table S2). *S. pneumoniae* AdhC also showed the highest similarity (90.9%) with that of *M. catarrhalis*, which is also in agreement with the phylogenetic relationship (Fig. S6). As seen with FghA, the AdhC proteins of closely related species diverged from that of *M. catarrhalis*.

### The putative *adhC–fghA* operon contributes to formaldehyde detoxification in *M. catarrhalis*

**Fig. 2:**
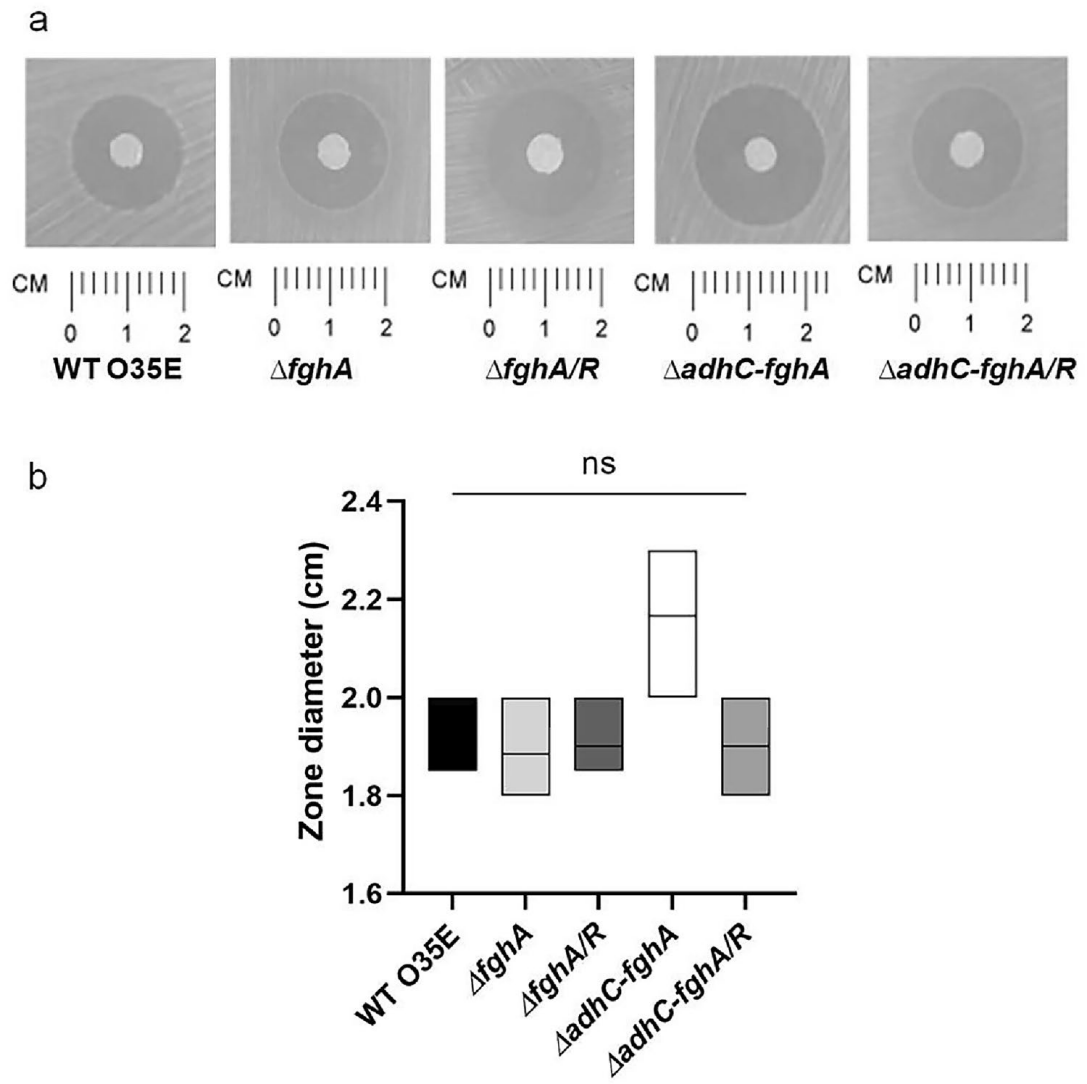
Loss of the *adhC–fghA* operon or *fghA* gene did not impair formaldehyde detoxification in *M. catarrhalis* when tested by disc diffusion assay. **A** Photographs of the zones of inhibition were taken using a gel documentation system (UVP) with a ruler under each strain that represents the scale in cm*.* **B** Box plot representing results of the formaldehyde disc diffusion assay, plotting the zone diameter obtained in cm. The data represent the mean of three independent experiments, and the bars span the difference between the minimum and maximum readings. The line inside the box represents the median. The graph was generated using GraphPad prism version 9.0.0 (GraphPad Software, San Diego, CA, USA). Statistical analysis of the data was done applying one-way ANOVA followed by Tukey’s multiple comparison test. The “ns” stands for “non-significant”

To investigate the possible role of *adhC* and *fghA* in formaldehyde detoxification in *M. catarrhalis*, deletion mutants lacking the *fghA* gene (*ΔfghA*) and both genes *fghA* and *adhC* (*ΔadhC-fghA*) were constructed, and along with their complemented mutants (*ΔfghA/*R and *ΔadhC-fghA/*R) were tested for their formaldehyde sensitivity compared to WT O35E using a disc diffusion susceptibility assay. There was no significant difference between the five strains in sensitivity to formaldehyde using this susceptibility assay (Fig. 2).

**Fig. 3:**
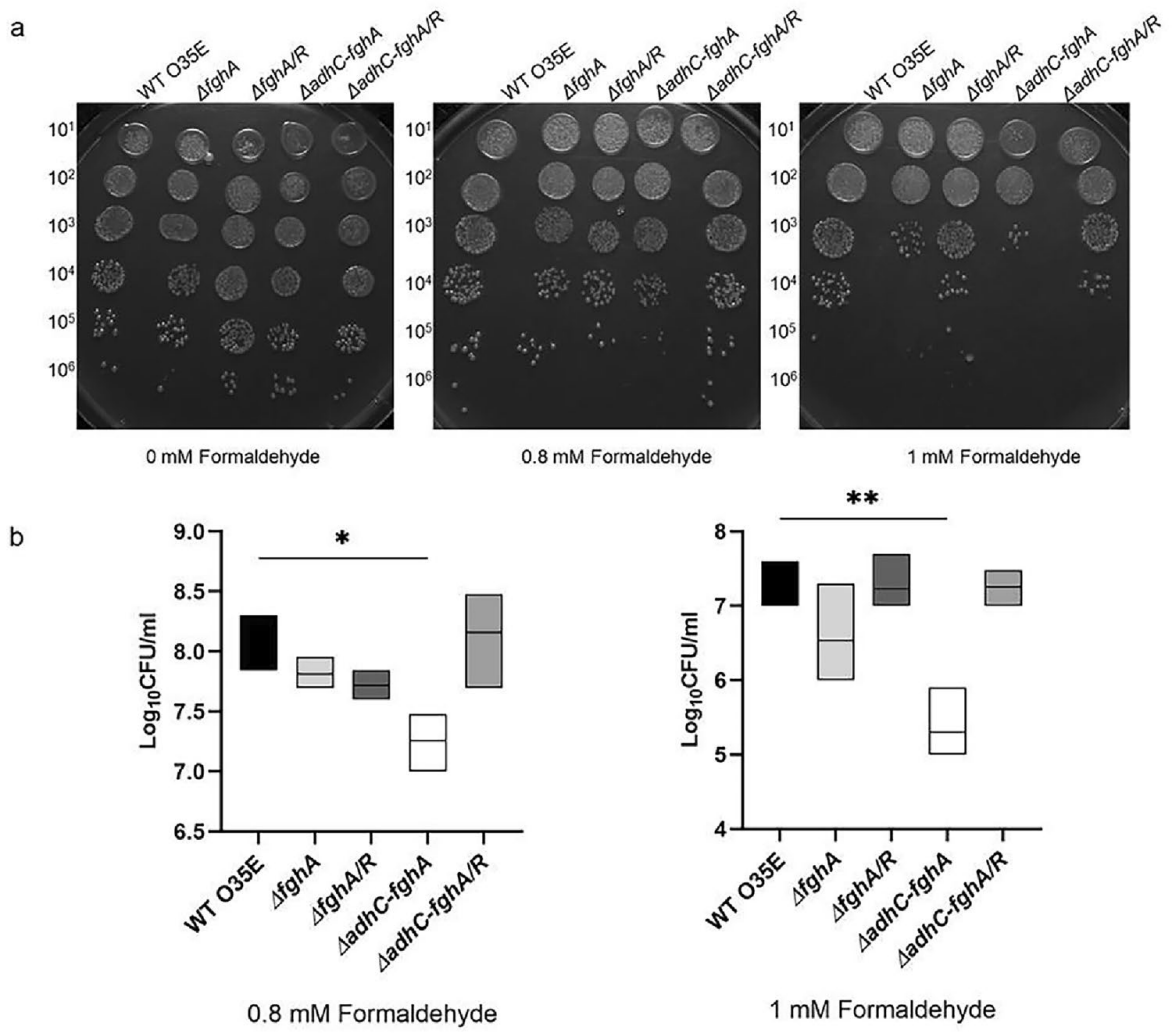
Loss of the *adhC–fghA* operon but not *fghA* impairs formaldehyde detoxification in *M. catarrhalis* when tested by serial dilution susceptibility assay. **A** Photographs of dilutions on TSA plates containing increasing concentrations of formaldehyde (0 mM, 0.8 mM, and 1 mM) were taken using a gel documentation system (UVP). **B** Box plot graphs of bacterial counts in CFU/mL with the different formaldehyde concentrations. The data represent the mean of three independent experiments, and the bars span the difference between the minimum and maximum readings. The line inside the box represents the median. The graphs were generated using GraphPad prism version 9.0.0 (GraphPad Software, San Diego, California USA). Statistical analysis of the data was done applying one-way ANOVA followed by Tukey’s multiple comparison test (*P ≤ 0.05), (**P ≤ 0.01). The * indicates that the difference is statistically significant

A more sensitive susceptibility assay was then performed by plating serial dilutions of the five strains on a solid medium containing increasing concentrations of formaldehyde (0, 0.8, and 1 mM). Especially at the 1 mM formaldehyde concentration, the growth of the *ΔadhC-fghA* was significantly attenuated (Fig. 3), while this growth defect was not observed with the rescue strain *ΔadhC-fghA/R* which reverted to the WT phenotype. Conversely, the single mutant *ΔfghA* and its complemented mutant *ΔfghA/R* did not display significant sensitivity to formaldehyde at any of the tested concentrations. It is worth mentioning here that all constructed mutants showed no growth defects in comparison to O35E when the growth was monitored logarithmically in plain TSB (Fig.S7).

Upon determination of the formaldehyde MIC for the five strains, results very similar to those observed above were obtained. For each of the four strains WT O35E, *ΔfghA, ΔfghA*/*R,* and *ΔadhC-fghA/R,* the MIC value was 1 mM. On the other hand, *ΔadhC-fghA* exhibited much lower MIC value of 7.8 µM.

### Loss of the formaldehyde detoxification system does not alter the protein expression profile in *M. catarrhalis* on SDS-PAGE

To investigate whether the loss of AdhC and FghA could alter the expression profile of some *M. catarrhalis* proteins, especially given the results of the predicted interactions obtained in silico from STRING which indicated the presence of a possible co-expression relationship between some of the protein pairs, the profiles following incubation with formaldehyde were visually assessed using SDS-PAGE. As the only co-expression interactions noticed in silico were between FghA with AdhC and FghA with Gdha, no prominent differences could be visually detected between the WT and any of the *ΔadhC-fghA* and the *ΔfghA* mutants or their complemented counterparts whether with or without formaldehyde (Fig.S8). This indicates that any differences that might exist are much more subtle to be detected using Coomassie blue staining of SDS-PAGE gels.

### The AdhC–FghA system is essential for pulmonary colonization by *M. catarrhalis*

**Fig. 4:**
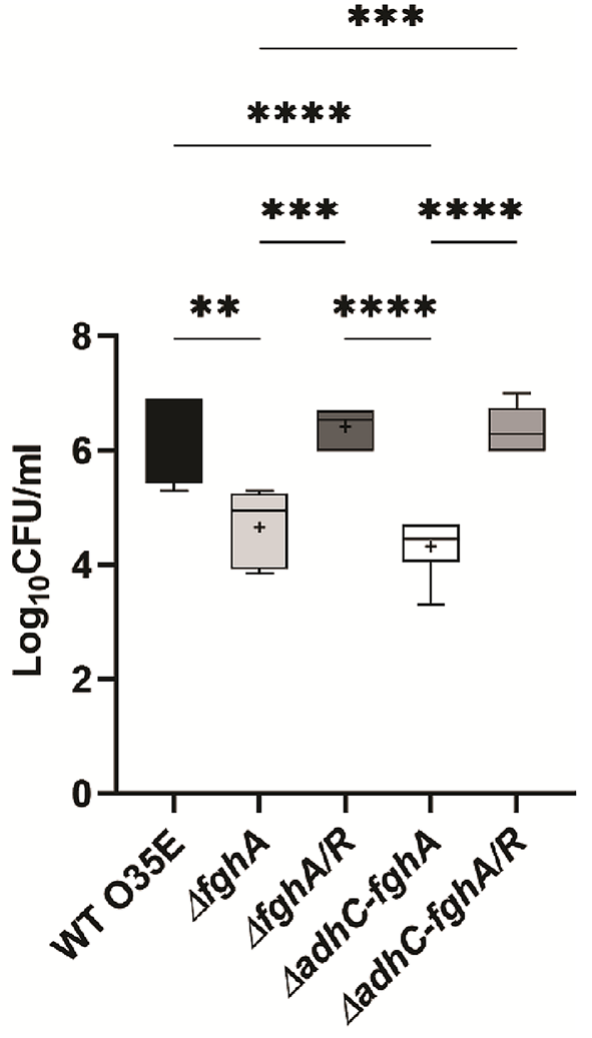
Loss of the *adhC–fghA* operon and *fghA* results in increased pulmonary clearance of *M. catarrhalis* in the murine pulmonary infection model. A box plot representing the colony counts in log_10_ CFU/mL, of *M. catarrhalis* WT O35E, *ΔfghA*, *ΔfghA*/R, *ΔadhC-fghA* double mutant, and *ΔadhC-fghA*/R as obtained from the lungs of infected mice. The bars span the difference between the minimum and maximum readings. The + sign represents the mean of the log_10_ CFU/mL. The graphs were generated using GraphPad prism version 9.0.0 (GraphPad Software, San Diego, CA, USA). Statistical analysis of data was performed by one-way ANOVA in GrapPad Prism. The * indicates that the difference is statistically significant as determined by Tukey’s multiple comparison test (**P ≤ 0.01), (***P ≤ 0.001), and (****P ≤ 0.0001)

To investigate whether the role of the AdhC–FghA system in formaldehyde detoxification would prove important to the fitness of *M. catarrhalis* in pulmonary infection, an *M. catarrhalis* pulmonary clearance model was conducted. Interestingly, mice were significantly more capable of clearing both *ΔfghA* and *ΔadhC-fghA* to a higher extent than WT O35E and the complemented mutants with an average one-log cycle change in the obtained colony counts (Fig. 4). These in vivo results indicate that AdhC–FghA system significantly contributes to the pathogenesis of *M. catarrhalis.*

## Discussion

Formaldehyde is highly cytotoxic to living organisms, so they need systems to detoxify formaldehyde to be able to survive. Several researchers investigated the formaldehyde detoxification mechanisms and the proteins involved to cope with this stress [4, 6, 7, 16, 18, 20]. However, the current study investigates the role of AdhC and FghA proteins in formaldehyde detoxification in *M. catarrhalis.*

*M. catarrhalis,* previously known as *N. catarrhalis,* resembles commensal *Neisseria* spp. in culture, phenotype, and ecological niche [44]*.* Therefore*, Neisseria* spp. previously characterized esterase protein EstD seemed to be a good template to search for similar formaldehyde detoxifying protein in *M. catarrhalis*. Our results indicated the high conservation of AdhC and FghA proteins across *M. catarrhalis* strains in addition to the species surveyed in silico in this study. These results could be attributed to the importance of the system for stress tolerance [4, 7, 20]. This was especially true for *S. pneumonia*e*,* which shared the highest similarity and evolutionary relationship with the *M. catarrhalis* proteins*.* The striking resemblance between these two organisms, which are considered two major causes of acute otitis media, could be due to their shared habitat. They share the same environment within the host, and this could have driven the development of comparable protective strategies to combat the same stresses encountered in the nasopharynx and upper respiratory tract [44].

The interaction network of AdhC and FghA with each other showed, most importantly, gene neighborhood, co-expression, and protein homology. This gave plausible evidence supporting the potential roles assigned to these proteins, and their functional relationship. The network also revealed the interaction by gene fusion between FghA with other proteins like that encoded by EA1_06621, a Gdsl-like lipase/ccyl hydrolase family protein. In addition, the MsrAB (EA1_03390), a trifunctional thioredoxin/methionine sulfoxide reductase a/b protein that has an important role as a repair enzyme for proteins that have been inactivated by oxidation [45] was predicted to have a gene neighborhood interaction with the FghA. The MsrAB mode of function is closely related to the mechanism of formaldehyde detoxification through the redox potential of glutathione. Another notable interaction was predicted with GdhA (EA1_02207), a NADP-specific glutamate dehydrogenase, that belongs to the Glu/Leu/Phe/Val dehydrogenases family. Glutamate metabolism plays an essential role in the synthesis of glutathione and Gdh-null mutants generally show a higher sensitivity to oxidative stress as well as a more rapid depletion of glutathione. These functions support the involvement of GdhA in the thiol-dependent pathway of formaldehyde detoxification [46, 47].

As expected, the Operon Mapper data indicated that *adhC* and *fghA* very likely constitute an operon. These findings are consistent with previous reports that also point out that these two genes form an operon in other species [4, 7, 14, 20, 43]. As per previous bioinformatic analysis, an attempt to confirm the function of *adhC* and *fghA* in formaldehyde detoxification in *M. catarrhalis* was designed. At first, a single mutant *ΔfghA* was constructed. There was no significant difference between the wild-type and the single mutant *ΔfghA* in the formaldehyde sensitivity tests, so a double mutant *ΔadhC-fghA* was constructed. A study conducted by Chen and co-workers mentioned that their single mutant of the *fghA* homolog was more sensitive to formaldehyde than their double mutant [20]. On the contrary, the current study shows that the increase in sensitivity is more significant in the double mutant *ΔadhC-fghA* than the single mutant *ΔfghA*. The high sensitivity to formaldehyde in the double mutant was expected as both proteins interact together in the formaldehyde detoxification pathway, while in the single mutant, the results showed that FghA is not essential on its own to detoxify formaldehyde. One speculation could be that, in the *adhC–fghA* mutant, due to the inactivation of the *adhC,* the accumulation of *S*-hydroxymethyl glutathione, the substrate of AdhC, is more toxic to the cells than the accumulation of *S*-formyl glutathione, the substrate of FghA, in the *fghA* mutant [4]. A complementary explanation for these results which would account for the non-toxicity of *S*-formyl glutathione could be attributed to the presence of another enzyme that participates with FghA, or completely replaces it, in the hydrolysis of *S*-formylglutathione to glutathione and formate. This explanation was also suggested by Harms and co-workers where they found that *fghA* mutant in *Paracoccus denitrificans* was able to grow on choline, a formaldehyde-generating substrate, which comes in agreement with our results [6]. Interestingly, during our bioinformatic analysis, we found that the two *E. coli* proteins YeiG and FrmB are two homologs for FghA of *M. catarrhalis* O35E. Gonzalez and co-workers mentioned that the simultaneous deletion of both *yeiG* and *frmB* genes is required to increase the sensitivity to formaldehyde, since the two proteins contribute to the detoxification of formaldehyde [7]. This could be the case in *M. catarrhalis*, with a structural homolog to FghA rather than a sequence homolog that is yet to be discovered, as none could be found in the proteome of *M. catarrhalis* using blast analysis. A study conducted by Potter and co-workers reported that each of the single mutants *ΔestD* and *ΔadhC* showed identical zones of inhibition to that of the WT by the disc diffusion formaldehyde sensitivity assay [17]. In the current work, the disc diffusion method could not differentiate the susceptibilities of the constructed mutants and their wild-type counterpart. However, upon using more quantitative methods, the differences in the formaldehyde susceptibilities were more prominent. It was found that a significant difference exists between the *adhC–fghA* mutant tested compared to the wild type which demonstrated that the system contributes to formaldehyde detoxification.

Previous studies have revealed the important role played by the *adhC–fghA* system in bacterial virulence [17, 20]*.* In the current work, both the *ΔfghA* and the *ΔadhC-fghA* mutants show marked decreased fitness in a pulmonary clearance model as is evidenced by the significantly lower colony counts retrieved from mice infected with these strains. This shows that regardless of its direct role in formaldehyde detoxification, FghA could contribute to the resistance to clearance of *M. catarrhalis* from the host cells by a mechanism that is yet to be elucidated. Interestingly, the obtained data come in accordance with a study which showed that an FghA homolog, EstD in *N. gonorrhoeae,* is necessary for bacterial growth in the host’s cervical epithelial cells, although it did not show a formaldehyde-sensitive phenotype using disc diffusion assay. The mentioned study reported that EstD had a potential role in the nitrosative stress defense system of *N. gonorrhoeae* which allows it to counteract the killing effect of nitric oxide [9] released by phagocytic cells in the inflammatory response to infection [17]. This shows that FghA could be involved in combating other kinds of stresses; hence, its role in pathogenesis warrants future studies. The significant increase in the pulmonary clearance extent of *ΔfghA* as well as *ΔadhC-fghA* compared to WT O35E reveals the necessity of both genes for survival in the respiratory tract of the host. Moreover, the current findings are similar to a previous study that pointed out to the possible important role the AdhC–EstD system plays in the survival and virulence of *N. meningitidis,* after observing that the *ΔadhC–estD* mutant is non-viable in experimentally induced biofilms [20].

To put this in a more biologically relevant context, Chen and co-workers reported that the concentration of formaldehyde in the blood of healthy individuals is estimated to be around 0.1 mM [4]. However, it is assumed that during inflammation, infection, and the associated respiratory burst, the localized concentration of formaldehyde would rise above the normal non-toxic levels. Furthermore, the complexity of the host response on the molecular level would certainly mean that other stresses are present and could contribute to the significantly higher clearance observed with the mutants.

## Conclusion

This study reports for the first time an *fghA* mutant and an *adhC/fghA* double mutant phenotypes in the emerging pathogen *M. catarrhalis.* The findings in this research indicate that the system plays a crucial role in formaldehyde detoxification and in lung colonization by *M. catarrhalis*. Moreover, these findings shed light on the importance of understanding the *adhC–fghA* system in *M. catarrhalis* to help in the potential development of novel therapeutics to combat infections caused by this emerging drug-resistant pathogen.

## Supplementary Information

Below is the link to the electronic supplementary material.

Supplementary file1 (PDF 2382 KB)
