# Supplementary material for: The Moraxella catarrhalis AdhC–FghA system is important for formaldehyde detoxification and protection against pulmonary clearance
Source: Med Microbiol Immunol. 2024 Mar 6;213(1):3. doi: 10.1007/s00430-024-00785-0 (PMC10917845; doi:10.1007/s00430-024-00785-0)
Supplement: Supplementary file 1 — Supplementary file1 (PDF 2382 KB) [file 430_2024_785_MOESM1_ESM.pdf]

The *Moraxella catarrhalis* AdhC- FghA system is important for formaldehyde detoxification and protection against pulmonary clearance

Dina Othman[1], Noha M. Elhosseiny[2], Wafaa N. Eltayeb[3], Ahmed S. Attia[2*],

[1]Graduate Program, Department of Microbiology and Immunology, Faculty of Pharmacy, Cairo University, Cairo, Egypt, 11562

[2]Department of Microbiology and Immunology, Faculty of Pharmacy, Cairo University, Cairo, Egypt, 11562

[3]Department of Microbiology, Faculty of Pharmacy, Misr International University, Cairo, Egypt,19648

Running title: Formaldehyde detoxification in *M. catarrhalis*

* Corresponding author

Address for correspondence:

Ahmed S. Attia, Ph.D.

Department of Microbiology and Immunology, Room #D404, Faculty of Pharmacy, Cairo University. Kasr El-Ainy Street, Cairo, Egypt, 11562.

Email: ahmed.attia@pharma.cu.edu.eg

Tel: +20-10-65344060

Fax: +20-2-23628246

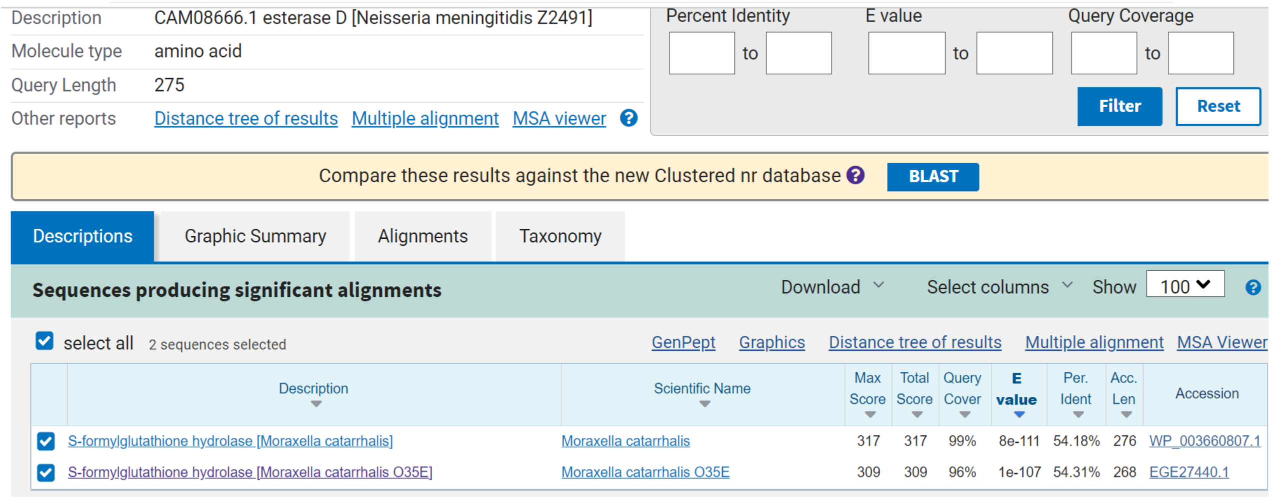

Description CAM08666.1 esterase D [Neisseria meningitidis Z2491]
Molecule type amino acid
Query Length 275
Other reports Distance tree of results Multiple alignment MSA viewer

Percent Identity to E value to Query Coverage to
Filter Reset

Compare these results against the new Clustered nr database BLAST

Descriptions Graphic Summary Alignments Taxonomy

Sequences producing significant alignments Download Select columns Show 100

select all 2 sequences selected GenPept Graphics Distance tree of results Multiple alignment MSA Viewer

Description	Scientific Name	Max Score	Total Score	Query Cover	E value	Per. Ident	Acc. Len	Accession
S-formylglutathione hydrolase [Moraxella catarrhalis]	Moraxella catarrhalis	317	317	99%	8e-111	54.18%	276	WP_003660807.1
S-formylglutathione hydrolase [Moraxella catarrhalis O35E]	Moraxella catarrhalis O35E	309	309	96%	1e-107	54.31%	268	EGE27440.1

Fig.S1 FghA in *Moraxella catarrhalis* O35E is a homolog for EstD in *Neisseria meningitides.* An illustration showing the BlastP results between *Neisseria meningitides* Z2491 (CAM08666.1) and other *M. catarrhalis* strains (taxid480). The Accession number, the query coverage and the percentage identity are illustrated. The figure is obtained from NCBI.

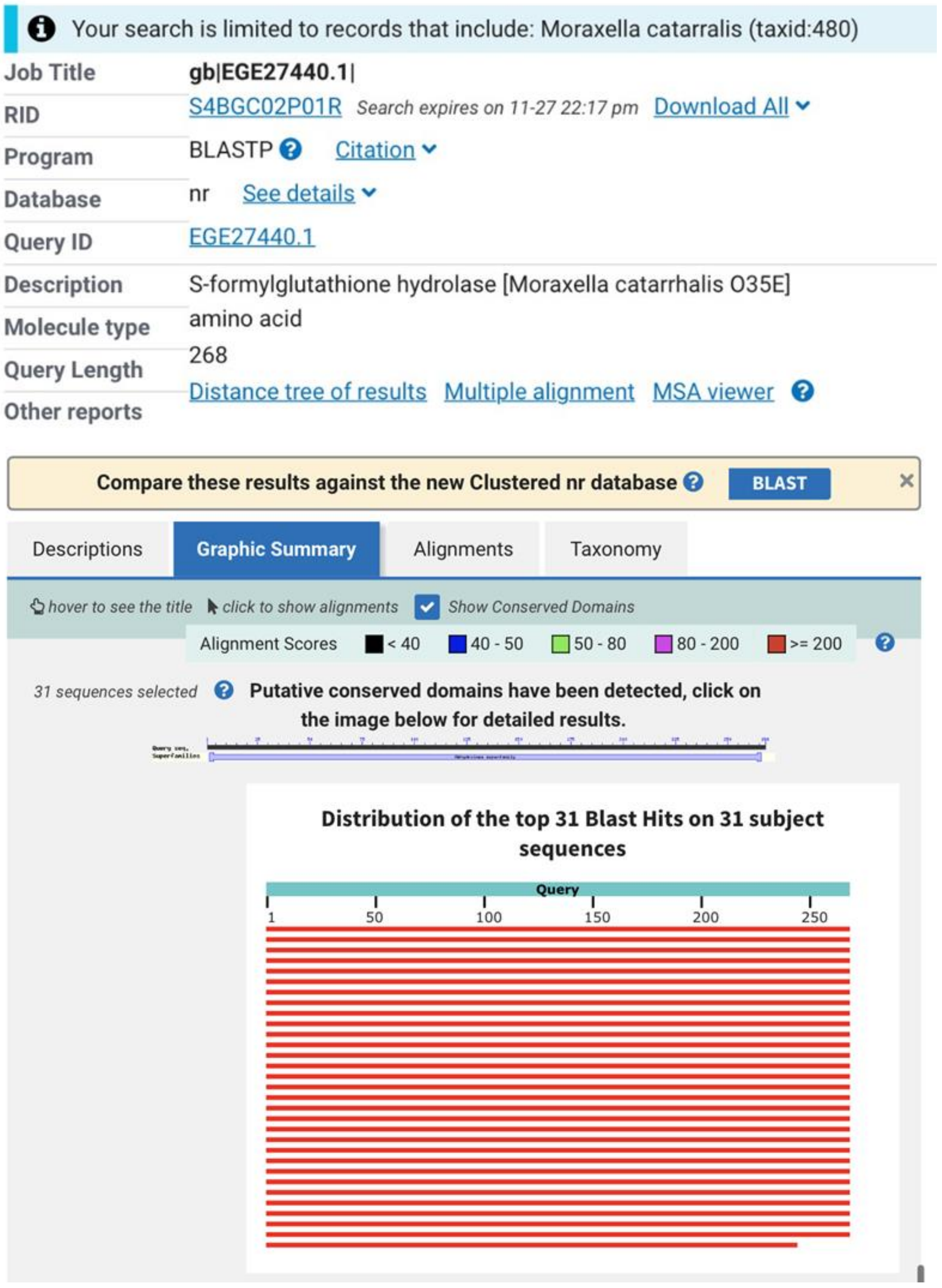

Fig.S2 FghA is conserved across *M. catarrhalis* strains. An illustration showing the BlastP results between *M. catarrhalis* O35E FghA and homologs in other *M. catarrhalis* strains (taxid480). The lines represent the extent of the query coverage as well as the degree of identity conservation. The figure is obtained from NCBI.

```
sp|P40363.1|SFGH_YEAST      --------MKVVKEFSVCGGRLIKLSHNSNSTKTSMNVNIYLPKHYYAQDF--PRNKRIP      50
OWB65897.1                  -----MSTFKIESEIASFGGKLLKLTHESPITNTKMDVNIYLPKKYFELKEKNSLEPELP      55
WP_011746369.1              ----MTLAYETVSENRSFGGIQGVYRHQSQATGTPMTFAIYLPPDA-------RH-GKVP      48
sp|P10768.2|ESTD_HUMAN      ------MALKQISSNKCFGGLQKVFEHDSVELNCKMKFAVYLPPKA-------ET-GKCP      46
WP_215354503.1              --------MKLIEQHQIFGGSQQVWAHHAQTLQCEMKFAVYLPDNR-------EN-QPLG      44
WP_005688077.1              --------MKLIEQHQIFGGSQQVWAHNAQTLQCEMKFAVYLPNNP-------EN-RPLG      44
pdb|4B6G|A                  ENLYFQGAMELIEQHQIFGGSQQVWAHHAQTLQCEMKFAVYLPNNP-------EN-RPLG      52
SGC68696.1                  --------MERIEHHVCFGGSQEVWRHHSAVTGTPMTFSVFLPPQA-------KT-EKCP      44
sp|P51025.2|SFGH1_ECOLI     --------MELIEKHVSFGGWQNMYRHYSQSLKCEMNVGVYLPPKA-------AN-EKLP      44
CAC87877.1                  ----MASGLSEIGSTKMFDGYNKRYKHFSETLGCSMTFSIYFPPSA-------SSSHKSP      49
EGE27440.1                  ----------------MFDGEHRRYRHVSKAVQSEMTFAVYLPKQA-------LKGACLP      37
WP_160545001.1              --------MKELSKNKLFGGWHKRFSHQSTSTGTEMTFAIYLPPQV-------EKEEKVP      45
sp|P33018.1|SFGH2_ECOLI     --------MEMLEEHRCFEGWQQRWRHDSSTLNCPMTFSIFLPPPR-------DH-TPPP      44
WP_053813516.1              ----MKDSLELIGSQKSFGGWHQRYRHFSRTLNCEMVFAIYLPPQA-------EPEAALP      49
                                               *      * :      * . :::*

sp|P40363.1|SFGH_YEAST      TVFYLSGLTCTPDNASEKAFWQFQADKYGFAIVFPDTSPRGDEVANDPEGSWDFGQGAGF      110
OWB65897.1                  VLLYLSGLTCTPNNASEKAFWQPYANEYGFAVVLPDTSPRGAGIEGE-DDSWDFGTGAGF      114
WP_011746369.1              VLWYLSGLTCTHENAMTKAGAQEWAAEYGIAVIFPDTSPRGEGVAN--DETYDLGQGAGF      106
sp|P10768.2|ESTD_HUMAN      ALYWLSGLTCTEQNFISKSGYHQSASEHGLVVIAPDTSPRGCNIKGE-DESWDFGTGAGF      105
WP_215354503.1              VIYWLSGLTCTEQNFITKSSFRRYAAEHQVIVVAPDTGPRGEQVPN--DAAYDLGQGAGF      102
WP_005688077.1              VIYWLSGLTCTEQNFITKSGFQRYAAEHQVIVVAPDTSPRGEQVPN--DAAYDLGQGAGF      102
pdb|4B6G|A                  VIYWLSGLTCTEQNFITKSGFQRYAAEHQVIVVAPDTSPRGEQVPN--DDAYDLGQSAGF      110
SGC68696.1                  VLYWLSGLTCNEQNFITKAGAQRYAAEHGLVIVAPDTSPRGSQVAD--DKEYDLGQGAGF      102
sp|P51025.2|SFGH1_ECOLI     VLYWLSGLTCNEQNFITKSGMQRYAAEHNIIVVAPDTSPRGSHVAD--ADRYDLGQGAGF      102
CAC87877.1                  VLYWLSGLTCTDENFIIKSGAQRAASTHGIALVAPDTSPRGLNVEGE-ADSYDFGVGAGF      108
EGE27440.1                  VLYYLSGLTCNDENFSTKSGAQQYAAKYGIILVIPDTSPRGETVPD--DEAYDLGQGAGF      95
WP_160545001.1              VLYFLSGLTCTDENFSTKAGAQQYAAKYGMALIMPDTSPRGEAVAD--DEAYDLGQGAGF      103
sp|P33018.1|SFGH2_ECOLI     VLYWLSGLTCNDENFTTKAGAQRVAAELGIVLVMPDTSPRGEKVAN--DDGYDLGQGAGF      102
WP_053813516.1              ALYWLSGLTCNDENFMQKAGAQRVAAELGLVLVAPDTSPRGSGVPGDPDGAWDFGLGAGF      109
                            .: :******. :*   *:  :  *    . :: ***.***  : .     :*:* .***

sp|P40363.1|SFGH_YEAST      YLNATQEPYAQHYQMYDYIHKELPQTLDSHFNKNGDVKLDFLDNVAITGHSMGGYGAICG      170
OWB65897.1                  YVDSIKEPWSKNYNMYSYILKDLLPNLKTDFKILN------FDKISITGHSMGGYGAMMF      168
WP_011746369.1              YVDATEAPWAPHFRMWHYVTHELPELVFNNFPLD-------REAQGITGHSMGGHGALTI      159
sp|P10768.2|ESTD_HUMAN      YVDATEDPWKTNYRMYSYVTEELPQLINANFPVD-------PQRMSIFGHSMGGHGALIC      158
WP_215354503.1              YLNATEQPWAANYQMYDYILNELPRLIEEHFPTN--------GKRSIMGHSMDGHGALVL      154
WP_005688077.1              YLNATEQPWATNYQMYDYILNELPDLIEANFPTN--------GKRSIMGHSMGGHGALVL      154
pdb|4B6G|A                  YLNATEQPWAANYQMYDYILNELPRLIEKHFPTN--------GKRSIMGHSMGGHGALVL      162
SGC68696.1                  YVNATQEPWKAHYQMYDYILNELHTLVSGHFGTS--------EKRGICGHSMGGHGALVL      154
sp|P51025.2|SFGH1_ECOLI     YLNATQAPWNEHYKMYDYIRNELPDLVMHHFPAT--------AKKSISGHSMGGLGALVL      154
CAC87877.1                  YLNATQEKWK-NWRMYDYVVKELPKLLSENFSQLD------TTKASISGHSMGGHGALTI      161
EGE27440.1                  YVNATASPWSVHYQMYDYIVDELPRLIEKNFNVG--------Q-KAICGHSMGGHGALQI      146
WP_160545001.1              YLNATEQPWAEHYRMYDYVVEELPALIEENFPVT--------DKRSIFGHSMGGHGALQI      155
sp|P33018.1|SFGH2_ECOLI     YLNATQPPWATHYRMYDYLRDELPALVQSQFNVS--------DRCAISGHSMGGHGALIM      154
WP_053813516.1              YLNATQEPWSAHYRMHDYVVNELPALIEAHFPVS--------QRRGISGHSMGGHGALVC      161
                            *:::    :  ::.*  *: .:*   :  .*              .* ****.* **:

sp|P40363.1|SFGH_YEAST      YLKGYSGKRYKSCSAFAPIVNPSNVPWGQKAFKGYLGEEKAQWEAYDPCLLIKNIRHVG-      229
OWB65897.1                  YLKNP--GYFKSCSAFSPISNPSKCPWGEKCFGNYLGDDKTKWLEYDPTELIKKFNNEGD      226
WP_011746369.1              AMTFP--ERYRSVSAFAPIAHPSESDWGRKQFAAYLGDDKAAWKRHDSTILMRE--KG--      213
sp|P10768.2|ESTD_HUMAN      ALKNP--GKYKSVSAFAPICNPVLCPWGKKAFSGYLGTDQSKWKAYDATHLVKSYPG---      213
WP_215354503.1              ALRNR--EHYQSVSAFSPILSPSLVPWGEKAFTAYLGKDREKWQQYDANSLIQQ--GY--      208
WP_005688077.1              ALRNR--ERYQSVSAFSPILSPSQVPWGEKAFTAYLGEDREKWQQYDASSLIQQ--GY--      208
pdb|4B6G|A                  ALRNQ--ERYQSVSAFSPILSPSLVPWGEKAFTAYLGKDREKWQQYDANSLIQQ--GY--      216
SGC68696.1                  ALRNP--DAFLSVSAFAPIVSPSQVPWGEKAFSAYLGEDKAAWEQYDAVKLIES--GH--      208
sp|P51025.2|SFGH1_ECOLI     ALRNP--DEYVSVSAFSPIVSPSQVPWGQQAFAAYLAENKDAWLDYDPVSLISQ--GQ--      208
CAC87877.1                  YLRNL--DKYKSVSAFAPITNPINCAWGQKAFTNYLGDNKAAWEEYDATCLISKYNN---      216
EGE27440.1                  GLKNP--DAYASISAFAPIVNPSKTPWGQKAFTAYLGEKESDWCEYDSTYLVAK--AT--      200
WP_160545001.1              GLKNY--EQYQSISAFSPIVNPSQVPWGQKAFTAYLGADKASWASYDSTHLLSQ--VD--      209
sp|P33018.1|SFGH2_ECOLI     ALKNP--GKYTSVSAFAPIVNPCSVPWGIKAFSSYLGEDKNAWLEWDSCALMYASNAQ--      210
WP_053813516.1              ALRNP--GRYLSLSAFAPICNPSDCPWGQKAFSRYLGEEPAAWREWDACALLET--AS--      215
                             :       : * ***:**  *    ** : *  **. .   *   *   *:

sp|P40363.1|SFGH_YEAST      ---DDRILIHVGDSDPFLE--EHLKPELLLEAVKATSWQDYVEIKKVHGFDHSYYFVSTF      284
OWB65897.1                  EKILPPILIHTGLNDPFYYRDFQLLPENLVAASKESKVK--VDVNLVDGYDHSYYFISSF      284
WP_011746369.1              --YPGEVLIDQGASDQFLD---LLKPEALAHAMAERRQP--GTFRMQQGYDHSYFFVQSF      266
sp|P10768.2|ESTD_HUMAN      --SQLDILIDQGKDDQFLL-DGQLLPDNFIAACTEKKIP--VVFRLQEGYDHSYYFIATF      268
WP_215354503.1              --KVQGMRIDQGLEDEFLP--TQLRTKDFIETCRAANQP--IDVRFHKGYDHSYYFIASF      262
WP_005688077.1              --KVQGIRIDQGLEDEFLP--TQLRTEDFIETCRVASQP--VDVRFHKGYDHSYYFIASF      262
pdb|4B6G|A                  --KVQGMRIDQGLEDEFLP--TQLRTEDFIETCRAANQP--VDVRFHKGYDHSYYFIASF      270
SGC68696.1                  --RVTDMLVDQGLADTFFS--KQLKTGLLKSACETHNIP--HTIRVHAGYDHSYYFIASF      262
sp|P51025.2|SFGH1_ECOLI     --RVAEIMVDQGLSDDFYA--EQLRTPNLEKICQEMNIK--TLIRYHEGYDHSYYFVSSF      262
CAC87877.1                  --LSATILIDQGENDQFYP--DQLLPSKFEEACKKVNAP--LLLRLHPGYDHSYYFIATF      270
EGE27440.1                  --KAPPIRIDQGLADDFYP--NQLQPEQFVAAAQENGIN--VELNLHDGYDHSYFFVASF      254
WP_160545001.1              --KARPILIDQGLDDDFYP--EQLQPESFSKAAADNNIE--VQLNLHAGYDHSYYFIASF      263
sp|P33018.1|SFGH2_ECOLI     --DAIPTLIDQGDNDQFLA--DQLQPAVLAEAARQKAWP--MTLRIQPGYDHSYYFIASF      264
WP_053813516.1              --ERLPILVDQGERDDFLA--VQLKPEALRRAAREAGHP--LELRLQPGYDHSYYFIASF      269
                                    :. *  * *      *    :               ..   *:****:*: :*

sp|P40363.1|SFGH_YEAST      VPEHAEFHARNLGLI--- 299
OWB65897.1                  TKDHAAHHAKYLGLSSKL 302
WP_011746369.1              MADHIRWHAERLG----- 279
sp|P10768.2|ESTD_HUMAN      ITDHIRHHAKYLNA---- 282
WP_215354503.1              IGEHIAYHAAFLK----- 275
WP_005688077.1              IGEHIAYHAAFLK----- 275
pdb|4B6G|A                  IGEHIAYHAAFLK----- 283
SGC68696.1                  IADHIAWHAARLNA---- 276
sp|P51025.2|SFGH1_ECOLI     IGEHIAYHANKLNMR--- 277
CAC87877.1                  IEDHISHHAQALEL---- 284
EGE27440.1                  IEVHIKFHANHLNA---- 268
WP_160545001.1              VEEHIAFHAKHLGIL--- 278
sp|P33018.1|SFGH2_ECOLI     IEDHLRFHAQYLLK---- 278
WP_053813516.1              IEDHLRHHARVLDG---- 283
                               *   **  *
```

Fig.S3 Alignment between the *M. catarrhalis* FghA with homologs from other bacterial and non-bacterial genera. Alignments of *Moraxella catarrhalis* O35E FghA (EGE27440.1) with homologs including *Streptococcus pneumoniae* (WP_160545001.1), *Neisseria gonorrhoeae* (WP_215354503.1), *Neisseria meningitides* Esterase D (pdb|4B6G|A), *Haemophilus influenzae* (WP_005688077.1), *E. coli* FrmB (sp|P51025.2|SFGH1), *Mycobacterium tuberculosis* FrmB (SGC68696.1), *E.coli* YeiG (sp|P33018.1|SFGH2), *Pseudomonas aeruginosa* (WP_053813516.1), *Paracoccus denitrificans* (WP_011746369.1), *Arabidopsis thaliana* (CAC87877.1), Human

Esterase D (sp|P10768.2|ESTD), *Candida boidinii* hypothetical protein (OWB65897.1), and *Saccharomyces cerevisiae (*sp|P40363.1|SFGH). The alignment was generated using Clustal W and the primary sequences were obtained from the NCBI. The following symbols denote the degree of residue conservation: (*) Asterisk (Asterisk indicates positions which have a single, fully conserved residue, (:) (colon) indicates conservation between groups of strongly similar properties, (.) (period) indicates conservation between groups of weakly similar properties.

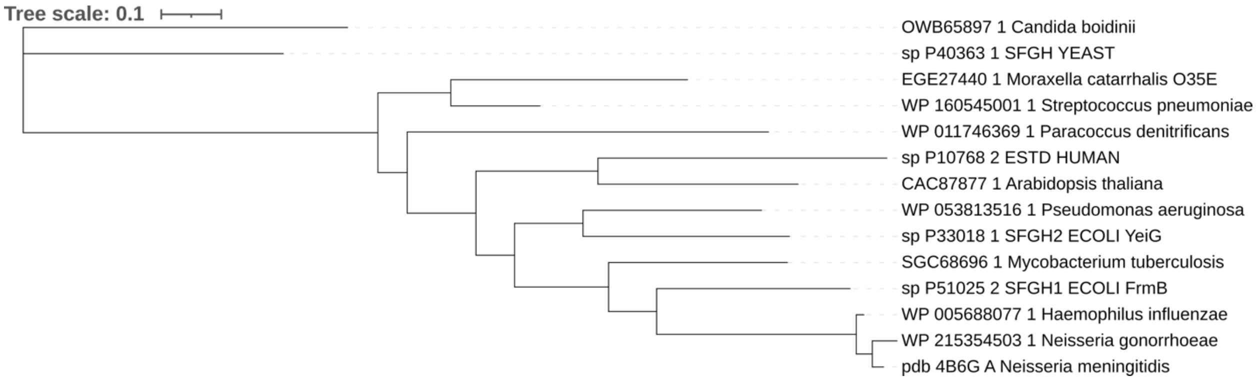

Fig.S4 Phylogeny of the *M. catarrhalis* FghA. A phylogenetic tree showing the evolutionary relationship between the *M. catarrhalis* O35E FghA and the previously studied proteins. The figure was generated using NGphylogeny web server, employing PhyML analysis using default parameters, and visualized using iTOL v6.

```
WP_011746366.1      -------------------MRTRAAVALEAGKPLEVMEVNLEGPKAGEVMVEIKATGICH      41
NP_308438.1         -------------------MKSRAAVAFAPGKPLEIVEIDVAPPKKGEVLIKVTHTGVCH      41
AKP14188.1          ----------MEMKQTDSTIKSRAAVAFAPNQPLQIVEIDVEMPRKDEVLIRNTHTGVCH      50
QEN74795.1          ----------MEMKQTDSSIKSRAAVAFAPNQPLQIVEIDVEMPRKGEVLIRNTHTGVCH      50
WP_204356019.1      -------------------MKSRAAVAFAPNQPLQIVEIDVEMPRKGEVLIRNTHTGVCH      41
MCF7750291.1        -------------------MKSRAAVAFGPGQPLKIVEIDVAPPKKGEVLVRITHTGICH      41
SGC68668.1          -------------------MKSRAAVAFGPGLPLEIVEIDVAPPKKGEVLVKISHTGVCH      41
NP_252319.1         ------------------MIKSRAAVAFAPNKPLEIVEVDVAPPQKGEVLVRIVATGVCH      42
EGE27439.1          ----------------MDFIKTRAAVAWAANEPLVIEELDLMPPQAGEVLVRIVATGVCH      44
WP_160545000.1      ----------------MEFIKTRAAVAWAPNEPLKIEELDLMMPQKGEVMVRITATGVCH      44
XP_019331123.1      ----------MSESTVGKPITCKAAVAWEAAKPLVIEDVTVAPPKAHEVRIKVLYSGVCH      50
NP_000662.3         --MANEVI------------KCKAAVAWEAGKPLSIEEIEVAPPKAHEVRIKIIATAVCH      46
NP_001190468.1      MATQGQVITCKGTFILDPRTLTISAVAYEPNKPLVIEDVQVAPPQAGEVRIKILYTALCH      60
                                           :***     ** : :: :  *:  ** :.   :.:**

WP_011746366.1      TDEFTLSGADPEGLFPSILGHEGAGVVVEVGPGVTSVKPGDHVIPLYTPECRQCASCLSG      101
NP_308438.1         TDAFTLSGDDPEGVFPVVLGHEGAGVVVEVGEGVTSVKPGDHVIPLYTAECGECEFCRSG      101
AKP14188.1          TDAFTLSGSDPEGVFPVVLGHEGAGVVVAVGEGVSSVKPGNHVIPLYTAECGECEFCLSG      110
QEN74795.1          TDAFTLSGSDPEGVFPVVLGHEGAGVVVAVGEGVSSVKPGDHVIPLYTAECGECEFCCSG      110
WP_204356019.1      TDAFTLSGSDPEGVFPVVLGHEGAGVVVAVGEGVLSVKPGDHVIPLYTAECGECEFCRSG      101
MCF7750291.1        TDAFTLSGDDPEGLFPAVLGHEGAGIVVEVGEGVTSVAPGDHVIPLYTAECGECLFCTSG      101
SGC68668.1          TDAYTLSGDDPEGLFPVVLGHEGAGVVVEVGEGVTSVKPGDHVIPLYTAECGECDFCTSG      101
NP_252319.1         TDAYTLSGQDSEGVFPCILGHEGGGIVEAVGEGVTSLQVGDHVIPLYTAECGKCKFCLSG      102
EGE27439.1          TDAYTLSGADPEGIFPCVLGHEGAGIVEAVGEGVTDFAVGDHVIPLYTAECGQCKMCLSN      104
WP_160545000.1      TDAYTLSGKDSEGVFPCVLGHEGAGIVEAIGEGVEGFEIGDHVIPLYTSECGKCQFCLSG      104
XP_019331123.1      TDAYTLSGVDPEGAFPVILGHEGAGIVESVGEGVTNVKPGDHVIALYTPECKECKFCKSG      110
NP_000662.3         TDAYTLSGADPEGCFPVILGHEGAGIVESVGEGVTKLKAGDTVIPLYIPQCGECKFCLNP      106
NP_001190468.1      TDAYTWSGKDPEGLFPCILGHEAAGIVESVGEGVTEVQAGDHVIPCYQAECRECKFCKSG      120
                    ** :* ** * ** ** :****..*:*  :* **  .  *: **  *  :* :*  * .

WP_011746366.1      KTNLCTAIRATQGQGLM-PDGTSRFSMLDGTPIFHYMGCSTFSNYTVLPEIAVAKVREDA      160
NP_308438.1         KTNLCVAVRETQGKGLM-PDGTTRFSY-NGQPLYHYMGCSTFSEYTVVAEVSLAKINPEA      159
AKP14188.1          KTNLCVSVRDTQGKGLM-PDGTTRFSY-QGQPIYHYMGCSTFSEYSVVAEVSLAKINPKA      168
QEN74795.1          KTNLCVSVRDTQGKGLM-PDGTTRFSY-QGQPIYHYMGCSTFSEYSVVAEVSLAKINPEA      168
WP_204356019.1      KTNLCVSVRDTQGKGLM-PDCTTRFSY-QGQPIYHYMGCSTFSEYSVVAEVSLAKINPEA      159
MCF7750291.1        KTNLCVAVRATQGKGVM-PDGTTRFSY-NGEPIYHYMGCSTFSEYTVVAEVSLAKINPDA      159
SGC68668.1          KTNLCVAVRETQGKGVM-PDGTSRFSY-NGQPLYHYMGCSTFSEYTVVAEVSLAKINPQA      159
NP_252319.1         KTNLCQAVRATQGKGLM-PDGTSRFSY-KGEPVFHYMGCSTFSEYTVLPEISLAKIPKDA      160
EGE27439.1          KTNLCSAVRETQGKGLM-PDGTVRFFK-EGEPIYHYMGTSTFSEYTVVSQYSLAKIQKDA      162
WP_160545000.1      KTNLCSAVRETQGRGLM-PDGTVRFFK-DGQPIYHYMGTSTFAEHTVVSEHSLVKIQEDA      162
XP_019331123.1      KTNLCGKIRATQGKGVM-PDGTPRFTC-KGKEILHFMGCSTFSQYTVVADISVVAINEKA      168
NP_000662.3         KTNLCQKIRVTQGKGLM-PDGTSRFTC-KGKTILHYMGTSTFSEYTVVADISVAKIDPLA      164
NP_001190468.1      KTNLCGKVRSATGVGIMMNDRKSRFSV-NGKPIYHFMGTSTFSQYTVVHDVSVAKIDPTA      179
                    *****  :* : * *:*  * . **   .*  : *:** ***::::*: : ::. :   *

WP_011746366.1      PFDKICYIGCGVTTGIGAVINTAKVEIGAKAVVFGLGGIGLNVLQGLRLAGADMIIGVDL      220
NP_308438.1         NHEHVCLLGCGVTTGIGAVHNTAKVQPGDSVAVFGLGAIGLAVVQGARQAKAGRIIAIDT      219
AKP14188.1          NHEQVCLLGCGVTTGIGAVHNTAKVQEGDSVAVFGLGAIGLAVVQGARQAKAGRIIAIDT      228
QEN74795.1          NHEQVCLLGCGVTTGIGAVHNTAKVQEGDSVAVFGLGAIGLAVVQGARQAKAGRIIAIDT      228
WP_204356019.1      NHEQVCLLGCGVTTGIGAVHNTAKVQEGDSVAVFGLGAIGLAVVQGARQAKAGRIIAIDT      219
MCF7750291.1        NPEQVCLLGCGVTTGIGAVHNTAKVQEGDSVAVFGLGGIGLAVIQGARQAKAGRIFAIDT      219
SGC68668.1          NAEQVCLLGCGVTTGIGAVHNTAKVQEGDSVAVFGLGGIGLAVVQGARQAKAGRIFAIDT      219
NP_252319.1         PLEKVCLLGCGVTTGIGAVLNTAKVEEGATVAIFGLGGIGLAAIIGAKMAKAARIIAVDI      220
EGE27439.1          PLEEVCLLGCGVTTGMGAVLKTAKVKPGDTVAIFGLGGIGLAAVIGARMAGAGRIIVVDI      222
WP_160545000.1      PLEEVCLLGCGVTTGMGAVKNTAKVEEGSSVAIFGLGGIGLAAIIGARMAKASRIIAIDT      222
XP_019331123.1      EFEKACLLGCGITTGYGAATITANVQEGDNVAVFGGGCVGLSVIQGCKERKVNKIILVDI      228
NP_000662.3         PLDKVCLLGCGISTGYGAAVNTAKLEPGSVCAVFGLGGVGLAVIMGCKVAGASRIIGVDI      224
NP_001190468.1      PLDKVCLLGCGVPTGLGAVWNTAKVEPGSNVAIFGLGTVGLAVAEGAKTAGASRIIGIDI      239
                      :. * :***: ** **.  **::: *   .:** * :** .  * :   .  *: :*

WP_011746366.1      NDDKKPMAEHFGMTHFINPKNCE--NVVQE-IVNLTKTPFDQIGGADYSFDCTGNVKVMR      277
NP_308438.1         NPKKFDLARRFGATDCINPNDYD--KPIKDVLLDI------NKWGIDHTFECIGNVNVMR      271
AKP14188.1          NPAKFELAKQCWCN----------------------------------------------      242
QEN74795.1          NPSKFELAKQFGATDCLNPNDYD--KPIKDVLLDI------NKWGIDHTFECIGNVNVMR      280
WP_204356019.1      NPAKFELAKQFGATDCLNPNDYD--KPIKDVLLDI------NKWGIDHTFECIGNVNVMR      271
MCF7750291.1        NPSKFELARQFGATDCVNPKDHD--KPIQQVIVEM------TTWGVDHTFECIGNVNVMR      271
SGC68668.1          NPSKFELAKQFGATDCINPNDYD--KPIQQVLVEM------TKWGVDHTFECIGNVNVMR      271
NP_252319.1         NPGKFDIARELGATDFINPKDYD--KPIQDVIVEL------TDGGVDYSFECVGNVQLMR      272
EGE27439.1          NEDKFAKAQELGATDCVNPNKFD--KPIQQVIIEM------TDGGVDFSFECIGNVDVMR      274
WP_160545000.1      NPEKFDKAKELGATDFVNPADFD--KQIQEVIIDM------TDGGVDYSFECIGNVEVMR      274
XP_019331123.1      NDKKEEWGKQFGATDFVNSTKLPEGTTIVDKLIEM------TDGGCDYTFDCTGNVNVMR      282
NP_000662.3         NKDKFARAKEFGATECINPQDFS--KPIQEVLIEM------TDGGVDYSFECIGNVKVMR      276
NP_001190468.1      DSKKYETAKKFGVNEFVNPKDHD--KPIQEVIVDL------TDGGVDYSFECIGNVSVMR      291
                    :  *   ...   .

WP_011746366.1      DALECTHRGWGQSIIIGVAPAGAEISTRPFQLVTGRVWKGTAFGGARGRTDVPQIVDWYM      337
NP_308438.1         AALESAHRGWGQSVIIGVAGSGQEISTRPFQLVTGRVWKGSAFGGVKGRSQLPGMVEDAM      331
AKP14188.1          ------------------------------------------------------------      242
QEN74795.1          QALESAHRGWGQSIIIGVAGAGQEISTRPFQLVTGRVWKGSAFGGVKGRSELPKMVEDSM      340
WP_204356019.1      QALESAHRGWGQSIIIGVAGAGQEISTRPFQLVTGRVWKGSAFGGVKGRSELPQMVEDSM      331
MCF7750291.1        AALECAHRGWGQSVIIGVAGSGQEISTRPFQLVTGRSWKGTAFGGVKGRSQLPGMVEDAM      331
SGC68668.1          SALESAHRGWGQSVIIGVAGAGKEISTRPFQLVTGRVWKGTAFGGVKGRTQLPGMVEDAM      331
NP_252319.1         AALECCHKGWGESVIIGVAGAGQEISTRPFQLVTGRVWRGSAFGGVRGRSELPSYVEKAQ      332
EGE27439.1          AALECCHKGWGESVIIGVAPAGAEISTRPFQLVTGRVWRGSAFGDVKGRSELPGIVSQYM      334
WP_160545000.1      TALECCHKGWGESIIIGVAPQGAEISTRPFQLVTGRVWRGSAFGGVKGKTQLPGIVKDYM      334
XP_019331123.1      NALEACHKGWGTSIIIGVAAAGKEISTRPFQLVTGRVWKGAAFGGVKGRSQLPGIVEDYL      342
NP_000662.3         AALEACHKGWGVSVVVGVAASGEEIATRPFQLVTGRTWKGTAFGGWKSVESVPKLVSEYM      336
NP_001190468.1      AALECCHKGWGTSVIVGVAASGQEISTRPFQLVTGRVWKGTAFGGFKSRTQVPWLVEKYM      351


WP_011746366.1      DGKIEIDPMITHTLSLDDINKGFDLMHAGESIRSVVLY--    375
NP_308438.1         KGDIDLEPFVTHTMSLDEINDAFDLMHEGKSIRTVIRY--    369
AKP14188.1          ----------------------------------------    242
QEN74795.1          KGDIELEPFVTHTMTLDQINKAFDLMHEGKSIRAVIHY--    378
WP_204356019.1      KGDIQLEPFVTHTMPLDQINEAFELMHKGKSIRTVIHY--    369
MCF7750291.1        KGDIELAPFVTHTMDLEQINDAFALMHEGKSIRSVVHY--    369
SGC68668.1          SGKIELAPFVTHTMELDKINEAFDLMHDGKSIRTVIHY--    369
NP_252319.1         KGEIPLDTFITHTMGLEDINEAFELMHEGKSIRTVIHY--    370
EGE27439.1          QGDFALSDFITHTMPLDQINAAFDLMHEGKSIRSVIHF--    372
WP_160545000.1      DGEFALSDFITHTMPLEQINDAFDLMHEGKSIRTVIHY--    372
XP_019331123.1      DGKLKVEEFITHREPLDKINTAFDEMHGGDCIRAVVSLW-    381
NP_000662.3         SKKIKVDEFVTHNLSFDEINKAFELMHSGKSIRTVVKI--    374
NP_001190468.1      NKEIKVDEYITHNLTLGEINKAFDLLHEGTCLRCVLDTSK    391
```

Fig.S5 Multiple sequence alignment for comparing the protein sequence of the AdhC in *M. catarrhalis* with the AdhC sequences obtained from other previously studied bacterial species. Alignments of *Moraxella catarrhalis* O35E S-(hydroxymethyl) glutathione dehydrogenase /class III alcohol dehydrogenase AdhC (EGE27439.1) compared with previously studied proteins including *Streptococcus pneumoniae* S-(hydroxymethyl)glutathione dehydrogenase/class III alcohol dehydrogenase (WP_160545000.1), *Pseudomonas aeruginosa* alcohol dehydrogenase (NP_252319.1), *Bacillus subtilis subsp. Subtilis* (S-(hydroxymethyl)glutathione dehydrogenase/class III alcohol dehydrogenase) MCF7750291.1, *Mycobacterium tuberculosis* Zinc-type alcohol dehydrogenase AdhD (SGC68668.1), *Neisseria meningitides* S-(hydroxymethyl)glutathione dehydrogenase/class III alcohol dehydrogenase (QEN74795.1), *Neisseria gonorrhoeae* S-(hydroxymethyl)glutathione dehydrogenase (AKP14188.1), *Haemophilus influenza* S-(hydroxymethyl)glutathione dehydrogenase/class III alcohol dehydrogenase (WP_204356019.1), *Escherichia coli* S-(hydroxymethyl)glutathione dehydrogenase (NP_308438.1), *Homo sapiens* alcohol dehydrogenase class-3 (NP_000662.3), *Arabidopsis thaliana* GroES-like zinc-binding dehydrogenase family protein (NP_001190468.1), *Candida albicans* bifunctional alcohol dehydrogenase/S-(hydroxymethyl)glutathione dehydrogenase (XP_019331123.1), *Paracoccus denitrificans* S-(hydroxymethyl)glutathione dehydrogenase/class III alcohol dehydrogenase (WP_011746366.1) Alignments were generated using Clustal W software and the primary sequences were obtained from the National Center for Biotechnology Information (NCBI). The following symbols denote the degree of residue conservation: (*)
(Asterisk) indicates positions which have a single, fully conserved residue, (:)

(colon) indicates conservation between groups of strongly similar properties, (.) (period) indicates conservation between groups of weakly similar properties.

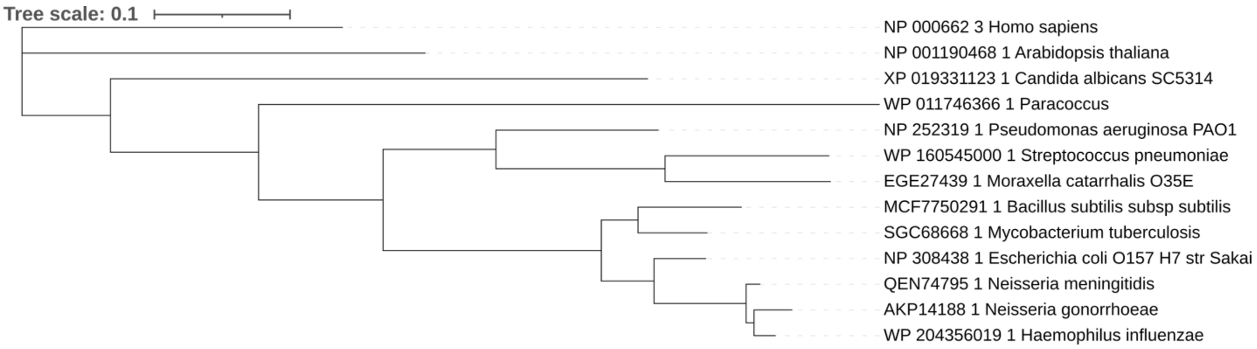

Fig.S6 A phylogenetic tree showing the phylogenetic relationship of *Moraxella catarrhalis* O35E S-(hydroxymethyl) glutathione dehydrogenase /class III alcohol dehydrogenase AdhC (EGE27439.1) with previously studied proteins. The figure was generated using NGphylogeny web server, employing PhyML analysis using default parameters, and visualized using iTOL v6.

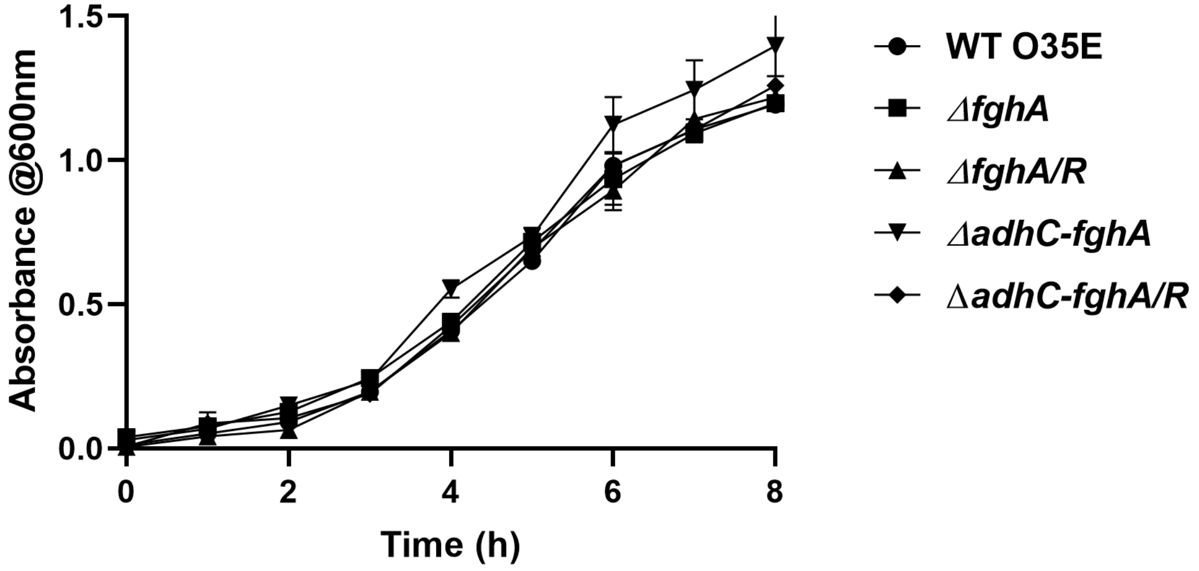

Fig.S7 Growth profile of *M. catarrhalis* WT O35E and the four *adhC-fghA* related constructs. A graph representing the growth kinetics of tested *M. catarrhalis* strains in TSB. The cultures were incubated with aeration at 37°C and shaking at 180 rpm. The growth was monitored by measuring the absorbance at 600 nm every hour. Each curve identification symbol is illustrated in the key in the figure. The graph was constructed by plotting the absorbance at 600 nm versus time in hours. The data represents the mean of three independent experiments and the error bars represent the SEM. The data was analyzed by one-way ANOVA. Tukey's multiple comparison test show no significant difference among SDs of the tested strains. The diagram was generated by GraphPad prism 9.0.0 (GraphPad Software, San Diego, California USA.).

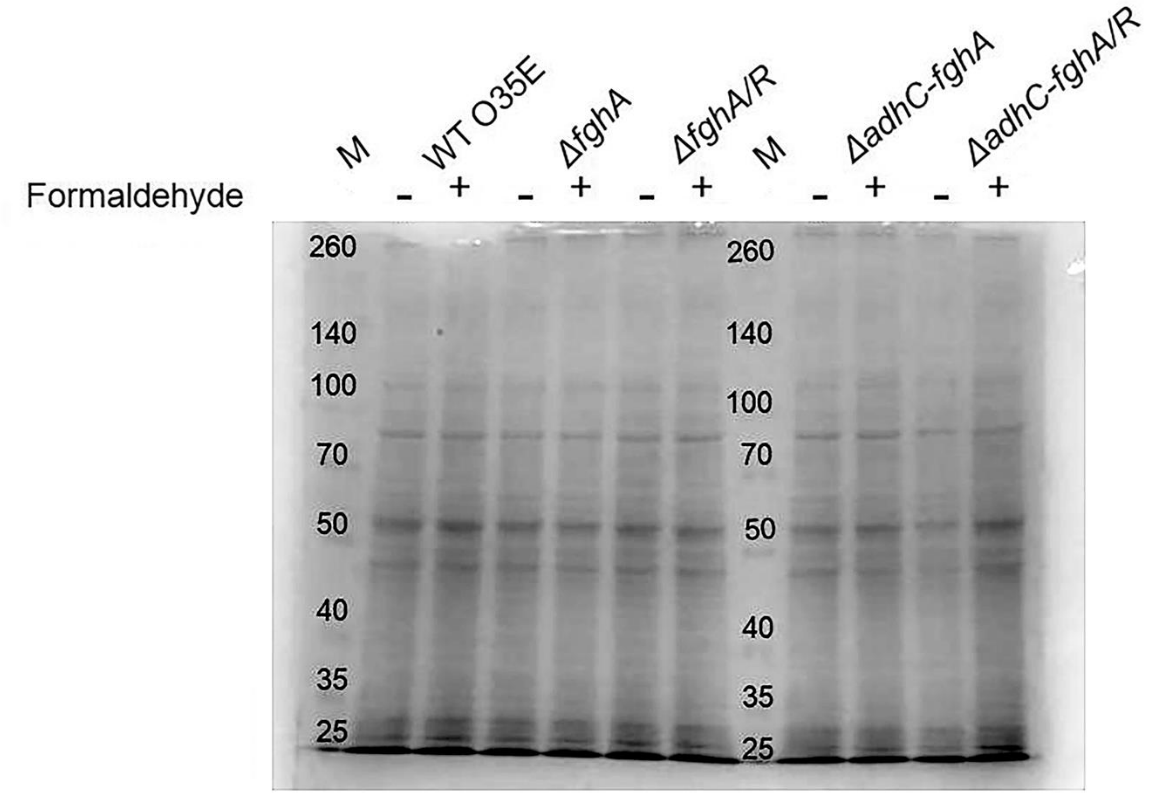

Fig.S8 Effect of formaldehyde stress on the protein expression of *M. catarrhalis*. The protein profiles of Wild type O35E WT, *ΔfghA* single mutant, *ΔfghA/R* complemented mutant, *ΔadhC-fghA* double mutant, and *ΔadhC-fghA/R* complemented mutant in the absence (-) and presence of 1 mM formaldehyde (+) as a stress factor were resolved using 10% SDS-PAGE and stained with Coomassie Brilliant Blue R250. (M) shows where the marker (Thermo Scientific Spectra Multicolor Broad Range Protein Ladder) was loaded. The numbers represent the molecular mass in kilodaltons. The photograph was captured using a gel documentation system (UVP).

Supplementary Table S1 The % identity and similarity of the FghA of *M. catarrhalis* O35E to that of different species calculated using EMBOSS Needle.

Name	Protein id	Source	% identity	%similarity
Streptococcus pneumoniae (S-formylglutathione hydrolase)	WP_160545001.1	Blast	65.8	77.7
Haemophilus influenzae (S-formylglutathione hydrolase)	WP_005688077.1	Blast	53.1	70.4
Mycobacterium tuberculosis (S-formylglutathione hydrolase FrmB)	SGC68696.1	Blast	53.4	70
Neisseria gonorrhoeae (S-formylglutathione hydrolase)	WP_215354503.1	Blast	50.2	69.3
E.coli (S-formylglutathione hydrolase YeiG)	P33018.1	[1]	54.1	69.2
Pseudomonas aeruginosa (S-formylglutathione hydrolase)	WP_053813516.1	Blast	55.5	68.6
*Neisseria meningitides (*Esterase D)	pdb|4B6G|A	[2]	50.9	68.4
E. coli (S-formylglutathione hydrolase FrmB)	P51025.2	[1]	52.5	68
Arabidopsis thaliana (S-formylglutathione hydrolase)	CAC87877.1	[1]	50	65
Paracoccus denitrificans (S-formylglutathione hydrolase)	WP_011746369.1	Blast	48.6	64.2
Human (Esterase D)	P10768.2	[1]	49.1	64.1
Candida boidinii (hypothetical protein)	OWB65897.1	Blast	43.7	57.6
*Saccharomyces cerevisiae (*S-formylglutathione hydrolase) ,	P40363.1	[1]	40.8	55.5

Supplementary Table S2 The % identity and similarity of the AdhC of *M. catarrhalis* O35E to that of different species calculated using the EMBOSS Needle.

Strain	Protein id	%identity to *M.catarrhalis* AdhC	% similarity to *M.catarrhalis* AdhC
Streptococcus pneumoniae (S-(hydroxymethyl)glutathione dehydrogenase/class III alcohol dehydrogenase)	WP_160545000.1	81.7	90.9
Pseudomonas aeruginosa (alcohol dehydrogenase)	NP_252319.1	76.1	85.2
Bacillus subtilis subsp. Subtilis (S-(hydroxymethyl)glutathione dehydrogenase/class III alcohol dehydrogenase)	MCF7750291.1	70.7	81.2
Haemophilus influenza (S-(hydroxymethyl)glutathione dehydrogenase/class III alcohol dehydrogenase)	WP_204356019.1	67.7	81.5
Escherichia coli (S-(hydroxymethyl)glutathione dehydrogenase)	NP_308438.1	68.5	81.2
Mycobacterium tuberculosis (Zinc-type alcohol dehydrogenase AdhD)	SGC68668.1	69.4	80.9
Neisseria meningitides (S-(hydroxymethyl)glutathione dehydrogenase/class III alcohol dehydrogenase)	QEN74795.1	67.5	80.4
Homo sapiens (alcohol dehydrogenase class-3)	NP_000662.3	64.2	79.7
Paracoccus denitrificans (S-(hydroxymethyl)glutathione dehydrogenase/class III alcohol dehydrogenase)	WP_011746366.1	58.8	74.7
Arabidopsis thaliana (GroES-like zinc-binding dehydrogenase family protein)	NP_001190468.1	58.3	72.6
Candida albicans (bifunctional alcohol dehydrogenase/S-(hydroxymethyl)glutathione dehydrogenase)	XP_019331123.1	58	71.9
Neisseria gonorrhoeae (S-(hydroxymethyl)glutathione	AKP14188.1	41.3	49.2

dehydrogenase)			

Supplementary Table S3: List of oligonucleotide primers used in this study

Primer name	Sequence	Description	Reference
DO001	5'-GTAGGCGAGGGCGTAAC-3'	Sequence (17 bp) forward primer binds at 936 bp upstream of the *fghA* ORF	This study
DO002	5'-AATcccgggAAAGGTCATCTCACTTT-3'	Sequence (26 bp) reverse primer binds at 66 bp after *fghA* ORF, XmaI restriction enzyme site is written in small caps	This study
DO003	5'-AATcccgggGCCTAAATTGGGATAAG-3'	Sequence (26 bp) forward primer binds at 801 bp downstream of the *fghA* ORF, XmaI restriction enzyme site is written in small caps	This study
DO004	5'-GTATCGTAGCAGCCTTTGGTATC-3'	Sequence (23 bp) reverse primer binds at 1807 bp downstream of the *fghA* ORF	This study
DO005	5'-ACCCCTGAAACGAATATGACCAAG-3'	Sequence (24 bp) screening forward primer binds at 86 bp upstream of the *adhC* ORF and 1241 bp upstream of the *fghA* ORF	This study
DO006	5'-ACTcccgggATGGAATTAATCAGCTGTCA-3'	Sequence (29 bp) forward primer binds at 24 bp upstream of the *fghA* ORF, XmaI restriction enzyme site is written in small caps	This study
DO007	5'-ACCGAggatccTTAGGCATTAAGATGATTTG-3'	Sequence (31 bp) reverse primer binds at 807 bp downstream of the *fghA* ORF. BamHI restriction enzyme site is written in	This study

		small caps	
DO008	5'-ATTGAggatccTCTGCAGCAAAAGAGTACGGT-3'	Sequence (32 bp) Forward primer binds at 329 bp upstream of the *adhC* ORF. BamHI restriction enzyme site is written in small caps	This study
DO009	5’-ACTcccgggCATCGGTTCCTTTGGTTAAT-3’	Sequence(29 bp) Reverse primer binds at 1 bp upstream of the *adhC* ORF. XmaI restriction enzyme site is written in small caps	This study
AA111	5’-GGGTGACTAACTAGGAGGAATAAAT-3’	Forward primer amplifies the promoterless kanamycin resistance (*kan*) cartridge from pUC18K3	[3]
AA116	5’-GGGTCGCATTATTCCCTCCAGGTA-3’	Reverse primer amplifies the promoterless kanamycin resistance (*kan*) cartridge from pUC18K3	[3]
Rpsl-5′	5′-TGGCGAACTCAAGCAAACAGC-3′	Forward primer used to generate a 3-kb amplicon containing the mutated *rpsL* gene plus flanking sequence	[4]
Rpsl-3′	5′-ACGCCACCAACAGCACAATAAACC-3′	Reverse primer used to generate a 3-kb amplicon containing the mutated *rpsL* gene plus flanking sequence	[4]

1. Gonzalez CF, Proudfoot M, Brown G, Korniyenko Y, Mori H, Savchenko AV, and Yakunin AF (2006) Molecular basis of formaldehyde detoxification. Characterization of two S-formylglutathione hydrolases from *Escherichia coli,* FrmB and YeiG. Journal of Biological Chemistry 281(20): 14514-22. https://doi.org/10.1074/jbc.M600996200
2. Chen NH, Counago RM, Djoko KY, Jennings MP, Apicella MA, Kobe B, and McEwan AG (2013) A glutathione-dependent detoxification system is required for formaldehyde resistance and optimal survival of *Neisseria meningitidis* in biofilms. Antioxidants & Redox Signaling 18(7): 743-755.
3. Attia AS, Sedillo JL, Wang W, Liu W, Brautigam CA, Winkler W, and Hansen EJ (2008) *Moraxella catarrhalis* expresses an unusual Hfq protein. Infection and Immunity 76(6): 2520-30. https://doi.org/10.1128/iai.01652-07
4. Attia AS and Hansen EJ (2006) A conserved tetranucleotide repeat is necessary for wild-type expression of the *Moraxella catarrhalis* UspA2 protein. Journal of Bacteriology 188(22): 7840-52. https://doi.org/JB.01204-06 [pii]

10.1128/JB.01204-06
